# Solitary fibrous tumor occurring in the parotid gland: a case report

**DOI:** 10.1186/s12907-017-0062-z

**Published:** 2017-11-21

**Authors:** Meryem Rais, Amine Kessab, Zahra Sayad, Sanae El Mourabit, Redallah Zrarqi, Salma Benazzou, Malik Boulaadas, Nadia Cherradi

**Affiliations:** 1 Department of Pathology, Hospital of Specialities, Rabat, Morocco; 2 Faculty of Medicine and Pharmacy of Rabat, Mohammed V University in Rabat, Rabat, Morocco; 3 Department of Plastic and Maxillofacial Surgery, Hospital of Specialities, Rabat, Morocco

**Keywords:** Solitary fibrous tumor, Parotid gland, Immunohistochemistry

## Abstract

**Background:**

Solitary fibrous tumor is an uncommon spindle cell neoplasm of unknown origin. It has been reported in many anatomic sites, with a rare occurrence in the head and neck region. Solitary fibrous tumors of the parotid gland are exceptional; their clinical and radiologic features are non specific, often mimicking more common salivary gland tumors. Pathologic examination and immunohistochemistry are required to make the correct diagnosis. The prognosis is favorable, with most tumors being benign, and complete surgical resection is the treatment of choice.

**Case presentation:**

We report the case of a 42-year-old man who presented with a painless mass involving the parotid gland. A parotidectomy was performed, and follow up was unremarkable. Gross examination showed a well circumscribed, firm tumor measuring 3,4 cm. Histologically, the tumor was composed of a spindle cell proliferation of variable cellularity, with staghorn vessels. A panel of immunohistochemical stains was performed, and confirmed the diagnosis of parotid gland solitary fibrous tumor.

**Conclusion:**

In this report we aim to increase awareness of this rare entity among clinicians and pathologists, and to emphasize the role of immunohistochemistry in confirming the diagnosis.

## Background

Solitary fibrous tumor (SFT) is a soft tissue neoplasm that was initially described in the pleura [1]. Since then, it has been reported in many anatomic sites, with about 6% developing in the head and neck [2]. However, SFT of the parotid gland is very rare, as only 29 cases were previously reported. Our study focuses on the clinical presentation, histopathological and immunohistochemical diagnosis, and review of the available literature regarding this rare tumor.

## Case presentation

**Fig. 1:**
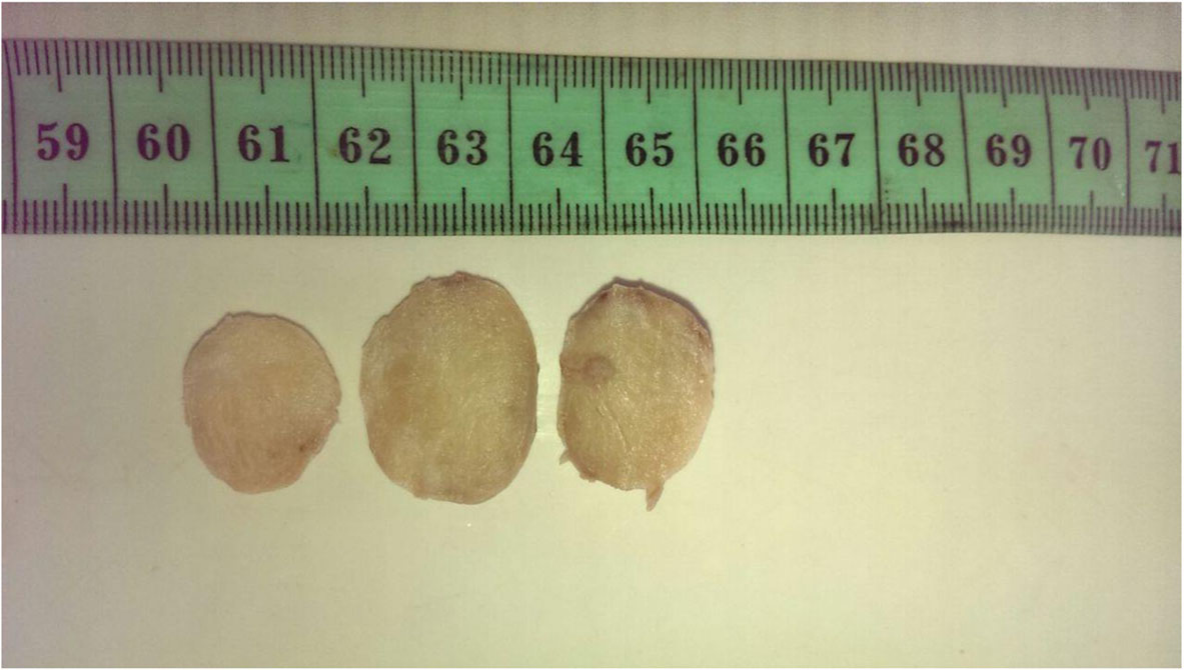
Macroscopic appearance of the tumor

**Fig. 2:**
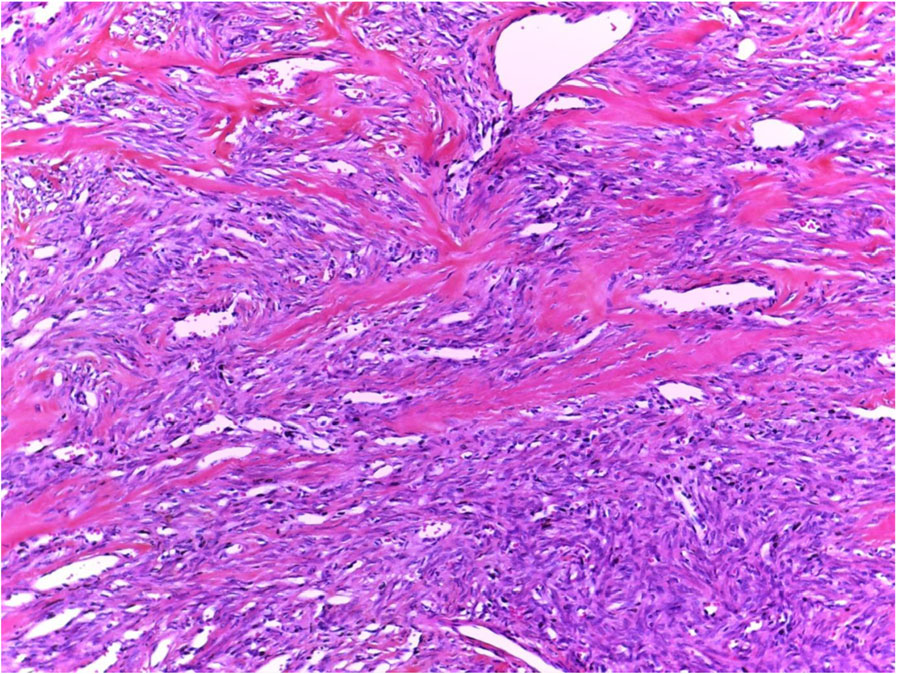
Hematoxilin & eosin (H&E) stain showing a spindle cell proliferation with “hemangiopericytoma like” vascularization and admixed ropy collagen (×100 magnification)

**Fig. 3:**
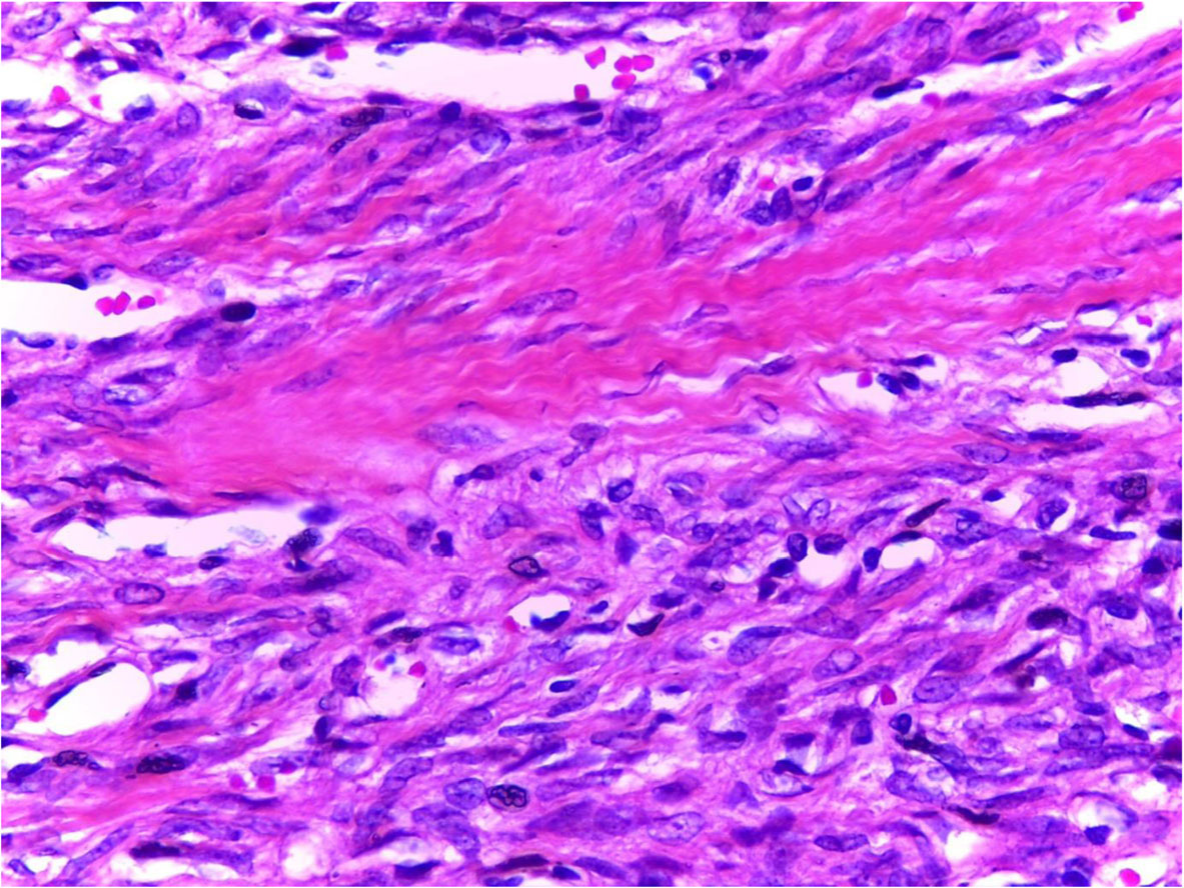
H&E stain showing an area of cellular spindle cell proliferation with mild atypia. Some lymphocytes are observed in the background, mitoses are not apparent (×200 magnification)

**Fig. 4:**
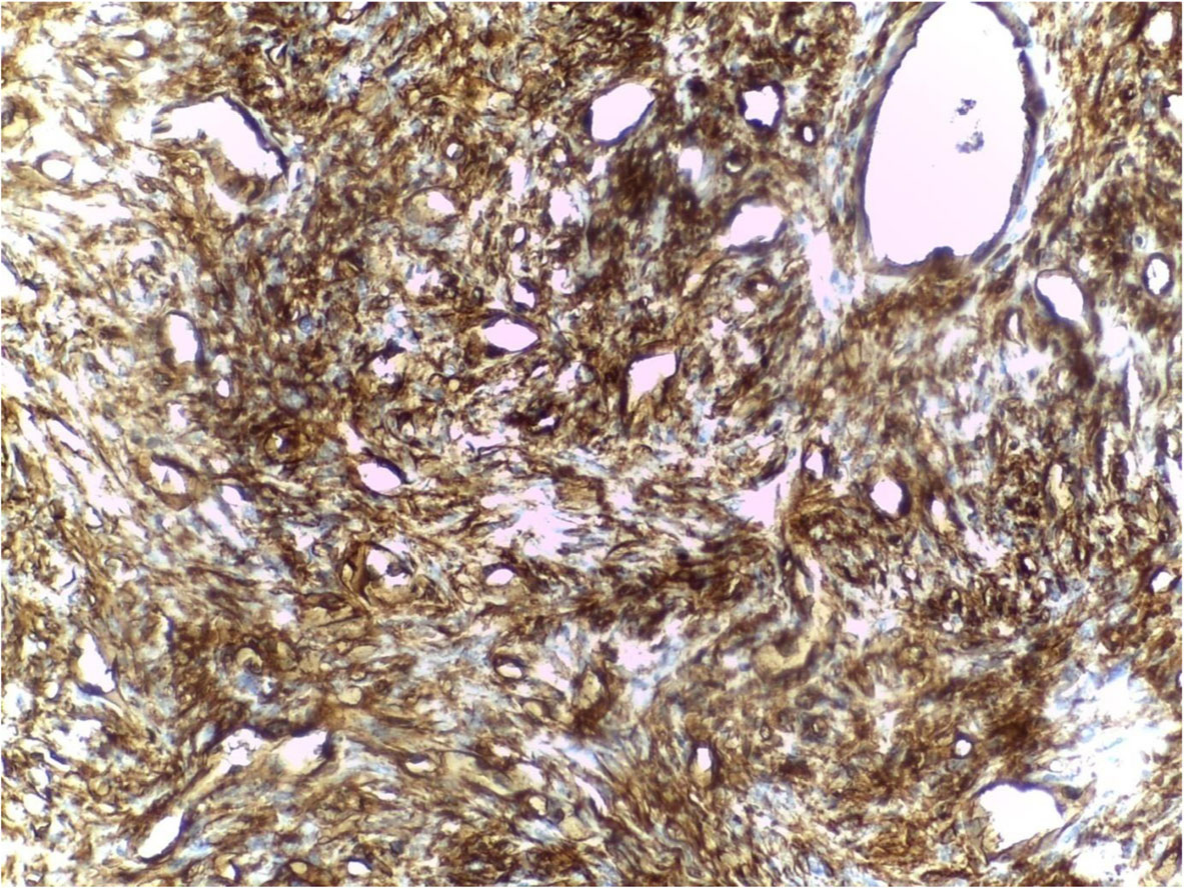
CD34 immunostain showing diffuse staining of the tumor cells

**Fig. 5:**
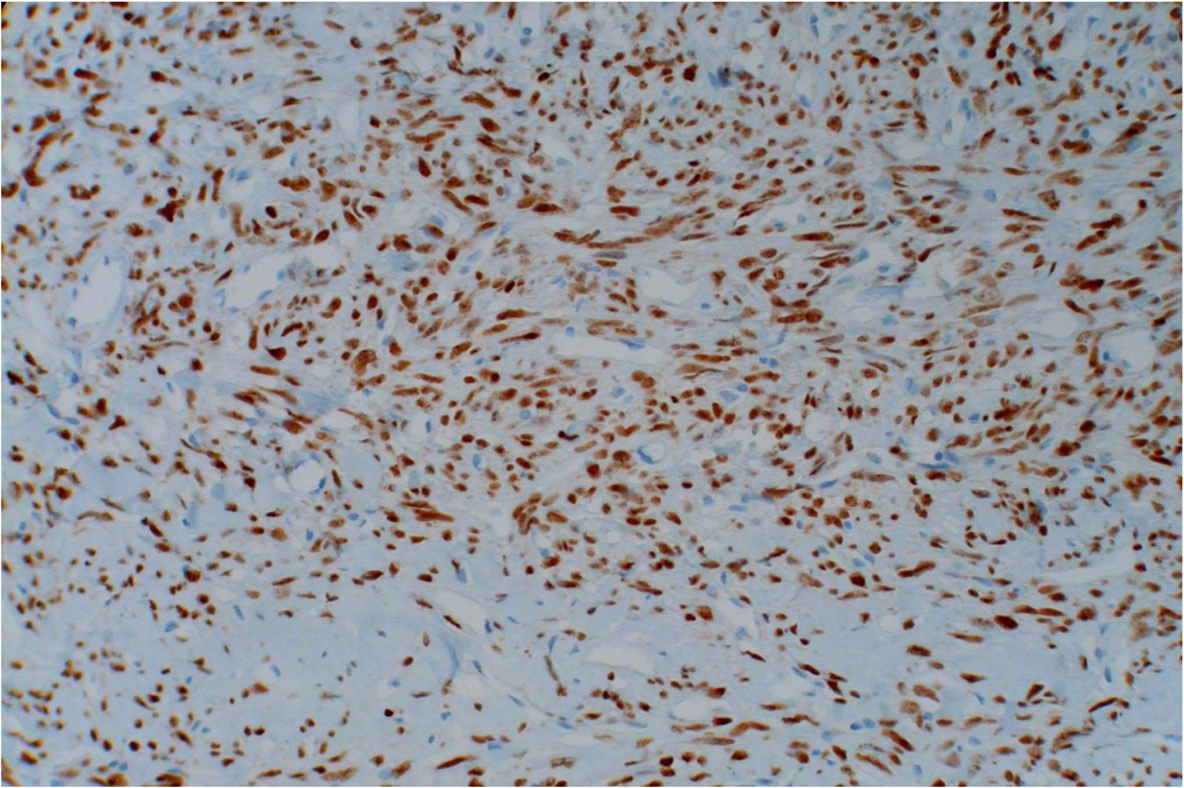
STAT6 immunostain showing diffuse staining of the tumor cells

A 42 year old man presented with the complaint of a slow growing, painless pretragal swelling of 5 years duration. The patient had no significant past medical or surgical history. The clinical examination found a 4 cm mass in the right parotid area. The overlying skin showed no sign of inflammation. The lesion was well circumscribed and soft in consistency. It was fixed to the underlying structures. There was no facial paralysis or cervical lymph node enlargement. Ultrasonography revealed a hypoechoic, moderately heterogeneous, well circumscribed, oval shaped mass, in the superficial lobe of the parotid gland. It had a moderate vascularity on Doppler, and measured 34x28x21 mm. These features were suggestive of a pleomorphic adenoma. A total parotidectomy was performed without complication. Macroscopically, the mass was well-defined and unencapsulated, it had a yellowish-tan color and a firm consistency (Fig. 1). Microscopic examination showed a well circumscribed proliferation of spindle cells arranged in a “paternless” pattern, with alternating hypo- and hypercellular areas separated by thick, hyalinized collagen with staghorn type vessels (Fig. 2). The nuclei showed mild to moderate atypia. (Figure 3). Mitotic figures were sparse (< 2 mitoses in 10 HPF). Necrosis was absent. On immunohistochemical studies, tumor cells were positive for CD 34 (Fig. 4) and STAT 6 (Fig. 5). They were negative with keratins, smooth muscle actin, S100 protein and CD31. A diagnostic of solitary fibrous tumor of the parotid gland was made. The patient has been followed-up for eleven months, with no signs of recurrence.

## Discussion

SFT is an exceedingly rare neoplasm in the parotid gland. In a case report and literature review in 2012, Bauer et al. [3] described 22 cases of this entity. Subsequently, 7 additional cases have been reported [4–10]. Therefore, including the case herein described, only 30 cases have been reported until now.

Solitary fibrous tumor (SFT) was described by Klemperer and Rabin in 1931 as a tumor of pleura [1]. Initially, this tumor was wrongly thought to be of mesothelial origin [11]. However, it was later demonstrated that SFT is a ubiquitous mesenchymal neoplasm, probably derived from adult stem mesenchymal cells [12]. In recent years, many studies using whole exome sequencing or RT-PCR, have identified a recurrent genetic mutation in SFTs [13, 14]. It is an intrachromosomal inversion: inv12(q13q13), resulting in a gene fusion: *NAB2-STAT6* which exhibits variable breakpoints and drives STAT6 nuclear expression [14].

Clinically, these tumors usually present as a palpable, painless well defined and slowly growing mass [3], as was described in our patient. This presentation is similar to other benign parotid tumors. In addition, radiographic findings are nonspecific. SFTs are typically hypoechogenic on ultrasonography. On computed tomography, they can be hypodense or hyperdense with respect to muscle. Magnetic resonance imaging usually shows an isointense mass on T1-weighted images and variable signal intensity on T2-weighted images [15]. Therefore, diagnosis of SFT is essentially based on histology and immunohistochemistry. Macroscopically, parotid SFT presents as firm, white-tan or gray, encapsulated, well-circumscribed lesions [3], similar findings were seen in our case. However, SFTs may be accompanied by bone destruction, normally without infiltration. This can be the result of a long-standing pressure effect [16]. Microscopically, these tumors consist of a patternless arrangement of spindle cells in a collagenous background with prominent blood vessels that result in a hemangiopericytoma-like pattern. There are usually alternating zones of hypercellularity and hypocellularity. The cell nuclei are round to oval, with open vesicular chromatin. Other features can be observed, such as stromal myxoid change, inflammatory cells and isolated multinucleated stromal tumor giant cells [3, 16]. Histological features suggesting malignancy include high mitotic rate (four or more mitoses in 10 high power fields), hypercellularity, moderate to marked atypia and nuclear pleomorphism, tumor necrosis and infiltrative borders [8]. These features were absent in the case of our patient. Even so, the histological appearance of SFT does not predict a malignant behavior with certainty [7].

Pathological differential diagnosis of SFT it extensive, and comprises cellular pleomorphic adenoma, myoepithelioma, schwannoma, neurofibroma, benign fibrous histiocytoma, nodular fasciitis, fibromatosis, myofibroblastoma, meningioma, fibrosarcoma, spindle cell squamous cell carcinoma, spindle cell melanoma, Kaposi sarcoma and monophasic synovial sarcoma [3]. For this reason, immunohistochemical examinations are required in order to confirm the diagnosis. SFTs are positive for CD34, CD99, Bcl2 and STAT6, but are negative with EMA and S100 protein. A strong nuclear diffuse STAT6 immunoreactivity has been shown to be highly sensitive and specific for SFTs [14]. This finding is relevant, as less than 10% of other spindle cell tumors are positive with STAT6, and do not show as diffuse and intense staining as in SFT [17]. In addition, STAT6 immunostaining presents an advantage in sparing a laborious RT-PCR, in which the difficulty results from important variability in both NAB2 and STAT6 breakpoints, requiring several RT-PCR assays to cover less prevalent variants of the mutation [14].

The treatment of SFTs is based on wide excision with negative surgical resection margins. Recurrence following a complete excision is rare [18]. Preoperative embolization may be performed in highly vascular tumors [19]. Since recurrence and metastasis can develop after several years, a long clinical and imaging regular follow-up is recommended [19].

## Conclusion

In summary, the current study presents a rare case of solitary fibrous tumor of the parotid gland occurring in a forty-two year-old man. As the clinical and radiologic features are non specific, the diagnosis is based on morphological and immunohistochemical analyses that allow exclusion of differential diagnoses.

## Abbreviation

SFT: Solitary fibrous tumor
